# Template nicking suppresses promoter-independent antisense transcription in IVT via R-loop-mediated strand displacement

**DOI:** 10.1093/nar/gkaf1536

**Published:** 2026-01-15

**Authors:** Goeun Lee, Junhyung Ryu, Jinmin Jang, Wooseong Noh, Dong-Eun Kim

**Affiliations:** Department of Bioscience and Biotechnology, Konkuk University, 120 Neundong-ro, Gwangjin-gu, Seoul 05029, Republic of Korea; Department of Bioscience and Biotechnology, Konkuk University, 120 Neundong-ro, Gwangjin-gu, Seoul 05029, Republic of Korea; Department of Bioscience and Biotechnology, Konkuk University, 120 Neundong-ro, Gwangjin-gu, Seoul 05029, Republic of Korea; Department of Bioscience and Biotechnology, Konkuk University, 120 Neundong-ro, Gwangjin-gu, Seoul 05029, Republic of Korea; Department of Bioscience and Biotechnology, Konkuk University, 120 Neundong-ro, Gwangjin-gu, Seoul 05029, Republic of Korea; R&D department, Uniwon PharmGene Inc., 120 Neungdong-ro, Gwangjin-gu, Seoul 05029, Republic of Korea

## Abstract

*In vitro* transcription (IVT) using T7 RNA polymerase is a cornerstone technology for synthetic messenger RNA (mRNA) production. However, a persistent challenge is the formation of immunogenic double-stranded RNA (dsRNA) byproducts, primarily arising from promoter-independent antisense transcription at free DNA ends. Here, we introduce the nicked low dsRNA template (NiLoT) strategy, a template engineering approach in which a single-strand nick is placed in the non-template DNA strand to promote R-loop formation and suppress antisense RNA synthesis. NiLoT significantly reduces dsRNA contamination without compromising RNA yield and enhances translational output while minimizing innate immune activation in human cells. This broadly applicable method improves IVT mRNA quality and supports more efficient and scalable RNA production.

## Graphical Abstract

**Graphical Abstract:**
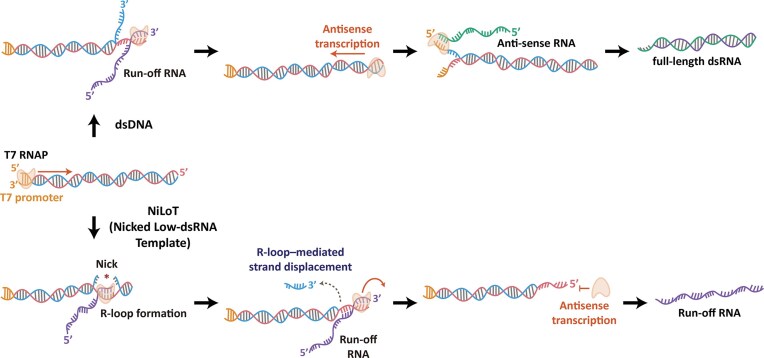

## Introduction

The clinical success of SARS-CoV-2 messenger RNA (mRNA) vaccines has accelerated the development of mRNA-based therapeutics and highlighted the importance of *in vitro* transcription (IVT) [1–4]. IVT using T7 RNA polymerase (T7 RNAP) enables the rapid, scalable, and sequence-specific synthesis of mRNA with high transcriptional efficiency [5]. However, a fundamental limitation of this technology is the co-production of immunostimulatory double-stranded RNA (dsRNA) byproducts, which compromise translational efficiency and provoke innate immune responses [6, 7].

Mechanistic investigations have revealed that the principal source of dsRNA in T7 RNAP-based IVT is promoter-independent antisense transcription initiated at blunt or overhang linear DNA termini lacking a T7 promoter sequence [8–11]. These aberrant antisense transcripts anneal with sense RNAs to form long, fully complementary dsRNA duplexes [8, 9]. These duplexes are recognized by intracellular pattern recognition receptors such as Toll-like receptor 3 (TLR3) [12], retinoic acid inducible gene I (RIG-I) [13, 14], and melanoma differentiation-associated protein 5 (MDA5) [15], and activate dsRNA-responsive enzymes including protein kinase R [16] and 2′-5′-oligoadenylate synthetase [17]. This recognition triggers type I interferon (IFN) production and translation inhibition, ultimately impairing mRNA stability and therapeutic performance [6, 7].

Several methods have been developed to reduce dsRNA formation in T7-based IVT by modifying reaction conditions or engineering T7 RNAP [9, 18]. While these strategies suppress dsRNA, they often reduce RNA yield, increase process complexity, or limit manufacturing scalability. For example, mutant T7 RNAP often exhibit reduced structural or thermal stability, complicating large-scale expression and purification. Adjusting MgCl_2_ concentrations [8] or adding chaotropic reagents [19] can lower dsRNA levels, but these approaches often reduce overall RNA yield and may require extensive re-optimization of reaction conditions [6, 20]. Importantly, most current strategies do not target the exposed DNA end that drives antisense transcription.

To overcome this limitation, we developed a template engineering strategy terms nicked low dsRNA template (NiLoT), which eliminates the double-stranded DNA (dsDNA) terminus required for antisense transcription by introducing a site-specific single-strand break (SSB) in the non-template strand. Our strategy builds on four prior insights: (i) nicked DNA templates promote R-loop formation during transcription [21, 22]; (ii) T7 RNAP can transcribe across SSBs without terminating elongation [23, 24]; (iii) strand displacement can be occurred by T7 RNAP [25, 26]; (iv) nascent RNA can displace the non-template strand through RNA:DNA hybrid-driven strand displacement [27]. These mechanisms suggested that a strategically positioned nick could promote R-loop formation and displace the downstream non-template strand, thereby preventing antisense transcription initiation at the terminus.

In this study, we demonstrate that NiLoT effectively reduces dsRNA levels without compromising RNA yield, remains compatible with various template formats and modified nucleotides, and improves both protein expression and immune tolerability in mammalian cells.

## Materials and methods

### *In vitro* transcription

IVT was performed using 50 nM of the indicated DNA template (sequences of oligonucleotide DNA shown in Supplementary Table S1) in a 20 µl reaction mixture containing 50 U of T7 RNA polymerase (Takara, Shiga, Japan, #2540A), 20 U of recombinant RNase inhibitor (Takara, #2313A), and 2 mM each ribonucleotide (rNTP; Promega, Madison, WI, USA, #P1221) in transcription buffer [40 mM Tris–HCl (pH 7.5), 8 mM MgCl_2_, 5 mM DTT, 2 mM spermidine]. For polymerase comparison experiments, three T7 RNA polymerases were used under identical reaction conditions; WT #1: Takara wild-type T7 RNA polymerase (Takara, #2540A), used throughout the study. WT #2: Thermo Fisher wild-type T7 RNA polymerase (Thermo Fisher Scientific, Waltham, MA, USA, #EP0111). Mutant #1: Takara PrimeCap™ engineered low-dsRNA variant (Takara, #2570A), optimized for reduced dsRNA production. Reactions were incubated at 37°C for 2 h, followed by treatment with 10 U of DNase I (New England Biolabs, Ipswich, MA, USA, #M0303S). RNAs were purified using the Monarch® RNA Cleanup Kit (New England Biolabs, #T2050L) according to the manufacturer’s instructions, and quantified using the Quant-iT™ RiboGreen RNA Assay Kit (Invitrogen, #R11490). Absolute RNA yields obtained under each transcription condition are summarized in Supplementary Table S2. IVT-synthesized RNA was analyzed by 1% agarose gel electrophoresis stained with GelRed (Biotium, Fremont, CA, USA, #41003), alongside the RiboRuler High Range RNA Ladder (Thermo Fisher Scientific, #SM1821), and further evaluated using an Agilent 4150 TapeStation system (Agilent Technologies, CA, USA) with RNA SreenTape according to the manufacturer’s protocol.

### Nuclease digestion assays

To assess RNA structure, 400 ng of IVT RNA was digested with S1 nuclease (Thermo Fisher Scientific, #EN0321) or RNase III (New England Biolabs, #M0245S). S1 nuclease digestion was performed using 1−6 µl of S1 nuclease (1:2500 dilution) in 1 × S1 buffer [40 mM sodium acetate (pH 4.5), 300 mM NaCl, 2 mM ZnSO_4_] at 37°C for 20 min. RNase III digestion was carried out using 1−6 µl of RNase III (1:2500 dilution) in buffer containing 50 mM Tris–HCl (pH 7.5), 50 mM NaCl, 1 mM DTT, and 10 mM MgCl_2_ at 37°C for 20 min. Reactions were stopped with 10 mM ethylenediaminetetraacetic acid (EDTA) and heat-inactivated at 70°C for 10 min.

### Preparation of RNA markers

Single-stranded RNA (ssRNA) markers were prepared by IVT RNA followed by cellulose-based removal of dsRNA [28]. 0.14 g of cellulose (Sigma–Aldrich, St. Louis, MO, USA, #C6288) slurry in 700 µl chromatography buffer [10 mM HEPES (pH 7.2), 0.1 mM EDTA, 125 mM NaCl, 16% ethanol] was loaded into Pierce™ spin columns (Thermo Fisher Scientific, #69725). RNA (100 μg in 500 µl chromatography buffer) was loaded onto the column and incubated for 30 min at RT. The columns were centrifuged at 16000 × *g* for 1 min at 4°C. Flowthrough was collected, and the purification was repeated twice. Final flowthrough was precipitated with isopropanol.

dsRNA markers were generated by annealing IVT-derived sense and antisense RNAs in hybridization buffer [30 mM HEPES (pH 7.2), 300 mM NaCl, 1 mM MgCl_2_] at 95°C for 3 min followed by slow cooling to 25°C for 30 min. dsRNA was recovered by isopropanol precipitation.

### Dot blot assay using dsRNA- or RNA:DNA hybrid-specific antibodies

To detect dsRNA, 200 ng of RNA in 10 µl was applied to a nylon membrane (Cytiva, #RPN303B) using a 96-well BioDot apparatus (Bio-Rad, #1706545). For detection of RNA:DNA hybrids, 5 µl of IVT reaction (without DNase I treatment) was spotted instead. Membranes were air-dried and blocked in TBS-T [20 mM Tris–HCl (pH 7.5), 150 mM NaCl, 0.1% Tween-20] containing 5% skim milk (BD Difco, #232100). Membranes were incubated overnight at 4°C with either the J2 monoclonal antibody specific for dsRNA (Scicons, #10010200) or the S9.6 antibody specific for RNA:DNA hybrids (Sigma–Aldrich, #MABE1095), both diluted 1:5000 in TBS-T containing 1% skim milk. After washing, membranes were incubated for 1 h at RT with horseradish peroxidase (HRP)-conjugated anti-mouse IgG (Santa Cruz Biotechnology, #sc-516102), also diluted 1:5000 in TBS-T with 1% skim milk. Signals were developed using the Immobilon® Western HRP substrate (Millipore, #WBKLS0500) and visualized using a G:BOX Chemi XL imaging system (Syngene). Signal intensities were quantified using ImageJ software.

### Preparation of long single-stranded DNA and partially duplex templates

#### Long single-stranded DNA 

dsDNA was generated by polymerase chain reaction (PCR) with a 5′-phosphorylated forward primer and non-phosphorylated reverse primer using Takara ExTaq® polymerase (Takara, #RR001A). The PCR product was purified (QIAquick PCR purification Kit, Qiagen, Venlo, The Netherlands, #28104) and subsequently digested with 10 U λ exonuclease (New England Biolabs, #M0262S) in buffer containing 67 mM glycine-KOH (pH 9.4), 2.5 mM MgCl_2_, and 50 μg/ml bovine serum albumin (BSA). The resulting single-stranded DNA (ssDNA) was purified using the APrep™ Gel DNA Kit (APBIO, Namyangju-si, Republic of Korea, #B1210). Strand specificity was validated by digesting 200 ng of ssDNA and dsDNA with 5 U S1 nuclease in 1 × S1 buffer or 5 U BsrGI-HF® (New England Biolabs, #R3575S) in NEB rCutSmart™ Buffer at 37°C for 20 min. Products were heat-inactivated at 80°C for 20 min and analyzed by 1% agarose gel electrophoresis (GelRed-stained) alongside the 100 bp DNA Ladder (New England Biolabs, #N0467S).

#### Partially duplex templates

Partially duplex templates were prepared by annealing the ssDNA with complementary 80 nt oligonucleotide encoding a T7 promoter (Supplementary Table S1) and analyzed by 4% native polyacrylamide gel electrophoresis (PAGE) with SYBR™ Gold (Invitrogen, #S11494) at 40 V for 2 h.

### Preparation of nicked low dsRNA template

To generate NiLoT DNA templates containing a site-specific nick on the non-template strand, three distinct methods were employed as described below.

#### PCR-based nicking

PCR products were gel-purified using the Aprep™ Gel DNA Kit and subsequently incubated with 50 U of Nt.Bpu10I (Thermo Fisher Scientific, #ER2011) in 1 × Buffer R [10 mM Tris–HCl (pH 8.5), 100 mM KCl, 10 mM MgCl_2_, 0.1 mg/ml BSA] at 37°C for 1 h. The reaction was heat-inactivated at 65°C for 20 min. Nicked DNA templates were purified using the QIAquick PCR Purification Kit.

#### Cas9 nickase-mediated nicking

For the experiment shown in Fig. 2B, 10 nM of gel-extracted PCR product was incubated with 100 nM of EnGen® Spy Cas9 Nickase (D10A mutant; New England Biolabs, #M0650T) and 150 nM target-specific sgRNA in 1 × NEB Buffer r3.1 at 37°C for 2 h. To facilitate ribonucleoprotein (RNP) complex formation, Cas9 D10A nickase and sgRNA were preincubated at 25°C for 10 min prior to DNA template addition. The D10A point mutation inactivates the RuvC nuclease domain while retaining HNH activity, thereby introducing a strand-specific nick exclusively on the non-template DNA strand. sgRNAs were designed using Benchling (https://benchling.com) such that the PAM sequence (5′-NGG-3′) was positioned on the template strand, ensuring that only the non-template strand is targeted for nicking. Five sgRNAs were selected to generate single-strand nicks at positions 97, 289, 417, 601, and 799 nucleotides from the non-template strand 3′ end. sgRNAs were synthesized *in vitro* using the EnGen® sgRNA Synthesis Kit (New England Biolabs, #E3322V), and their target sequences are listed in Supplementary Table S1. After the nicking reaction, samples were sequentially treated with (i) 0.1 U of Thermolabile Proteinase K (New England Biolabs, #P8111S), (ii) 0.1 µg of RNase A (Qiagen, #19 101), and (iii) a second Thermolabile Proteinase K treatment to ensure removal of protein and RNA contaminants. Final purification was performed using the QIAquick PCR Purification Kit (Qiagen), following the manufacturer’s instructions.

#### Plasmid-based nicking

Plasmids were first linearized using HindIII-HF® (New England Biolabs, #R3104S), purified with the QIAquick PCR Purification Kit, and subjected to nicking with Nt.Bpu10I under the same conditions as described above. The nicked plasmid DNA was subsequently re-purified.

#### Nick validation assay

To confirm successful nicking, 100 ng of each NiLoT sample was digested with 5 U of S1 nuclease in 1 × S1 buffer at 37°C for 1 h. Reaction products were analyzed by 1% agarose gel electrophoresis alongside a 100 bp DNA ladder.

### RNase H treatment

To confirm S9.6 specificity, IVT solution (without DNase I treatment) was treated with 10 U RNase H (New England Biolabs, #M0297S) at 37°C for 30 min. RNA was purified with the Monarch® RNA Cleanup Kit and used for dot blot.

### S1 nuclease protection assay

To detect strand displacement, IVT products were purified using Monarch® RNA Cleanup Kit and incubated with 200 nM 30 nt FAM-labeled probe (Supplementary Table S1) in hybridization buffer at 37°C for 2 h. Samples were digested with 1 U S1 nuclease in 1 × S1 buffer at 37°C for 20 min, terminated with 10 mM EDTA, and heat-inactivated at 70°C for 10 min. Samples were denatured with RNA Gel Loading Dye (2 ×, Thermo Fisher Scientific, #R0641) at 95°C for 3 min and run on 10% denaturing PAGE at 120 V for 30 min. FAM signal was visualized by UV illuminator. A fully complementary 30 nt oligonucleotide (Supplementary Table S1) was used as a positive control. Signal intensities were quantified using ImageJ.

### eGFP mRNA synthesis and polyadenylation

eGFP mRNA was transcribed using dsDNA or NiLoT with either unmodified or modified NTP (pseudouridine, Ψ; N1-methylpseudouridine, m^1^Ψ; 5-methylcytidine, m^5^C; N6-methyladenosine, m^6^A; TriLink, San Diego, CA, USA, #N-1019, #N-1081, #N-1014, #N-1013). For co-transcriptional capping, 1.6 mM CleanCap® AG (TriLink, #N-7113) was included in the IVT reaction. After DNase I digestion and purification with Monarch® RNA Cleanup Kit, 10 μg RNA was polyadenylated using 5 U E. coli poly(A) Polymerase (New England Biolabs, #M0276L) at 37°C for 30 min, then re-purified.

### Cell culture and transfection

HEK293T cells (ATCC, Manassas, VA, USA) were cultured in DMEM (WelGene, Gyeongsan, Republic of Korea, #LM001-07) with 10% FBS (Gibco, Grand Island, NY, USA, #A5256701) and 1% penicillin/streptomycin (Biowest, Nuaillé, France, #L0022) at 37°C and 5% CO_2_. THP-1 cells (Korean Cell Line Bank, Seoul, Republic of Korea) were maintained in RPMI1640 (Biowest, #L0500) containing 10% heat-inactivated FBS, 0.05 mM 2-mercaptoethanol (Gibco, #21 985 023), and 1% penicillin/streptomycin at 37°C and 5% CO_2_. HEK293T cells were transfected at 80% confluency in 24-well plates with 500 ng eGFP mRNA using Lipofectamine™ 3000 (Thermo Fisher Scientific, #L3000001) in Opti-MEM (Gibco, #31 985 070) for 4 h. THP-1 cells were seeded at 1.5 × 10^5^ cells per well in 24-well plates with 500 ng eGFP mRNA using Lipofectamine™ 3000 in RPMI1640 containing 5% heat-inactivated FBS and 0.05 mM 2-mercaptoethanol for 4 h. After transfection, the media were replaced with complete growth media, and cells were analyzed after 18 h by flow cytometry (FACSCalibur, BD Biosciences, San Jose, CA, USA) and fluorescence microscopy (Invitrogen).

### IFN-β detection

For the experiment shown in Fig. 5C and F, HEK293T and THP-1 cells were transfected as above. For the experiment shown in Supplementary Fig. S8B, THP-1 cells were transfected with 100 ng of IVT RNAs subjected to the following nuclease treatments: (i) DNase I^−^/RNase H^−^, (ii) DNase I^−^/RNase H^+^, (iii) DNase I^+^/RNase H^−^, (iv) DNase I^+^/RNase H^+^, and (v) DNase I^+^/RNase H^−^/RNase III^+^. After 18 h, 500 µl of supernatant was analyzed using a human IFN-β ELISA Kit (Cusabio, Wuhan, China, #CSB-E09889h). Absorbance was measured at 450/560 nm using VICTOR X3 plate reader. High molecular weight (HMW) Poly(I:C) (InvivoGen, San Diego, CA, USA, #tlrl-pic) was used as a positive control.

### Statistical analysis

Statistical analysis was conducted using GraphPad Prism version 8.0.2. Data are presented as mean ± SD from n = 3 biological replicates.

## Results

### Antisense transcription generates dsRNA contaminants during IVT

**Figure 1.**
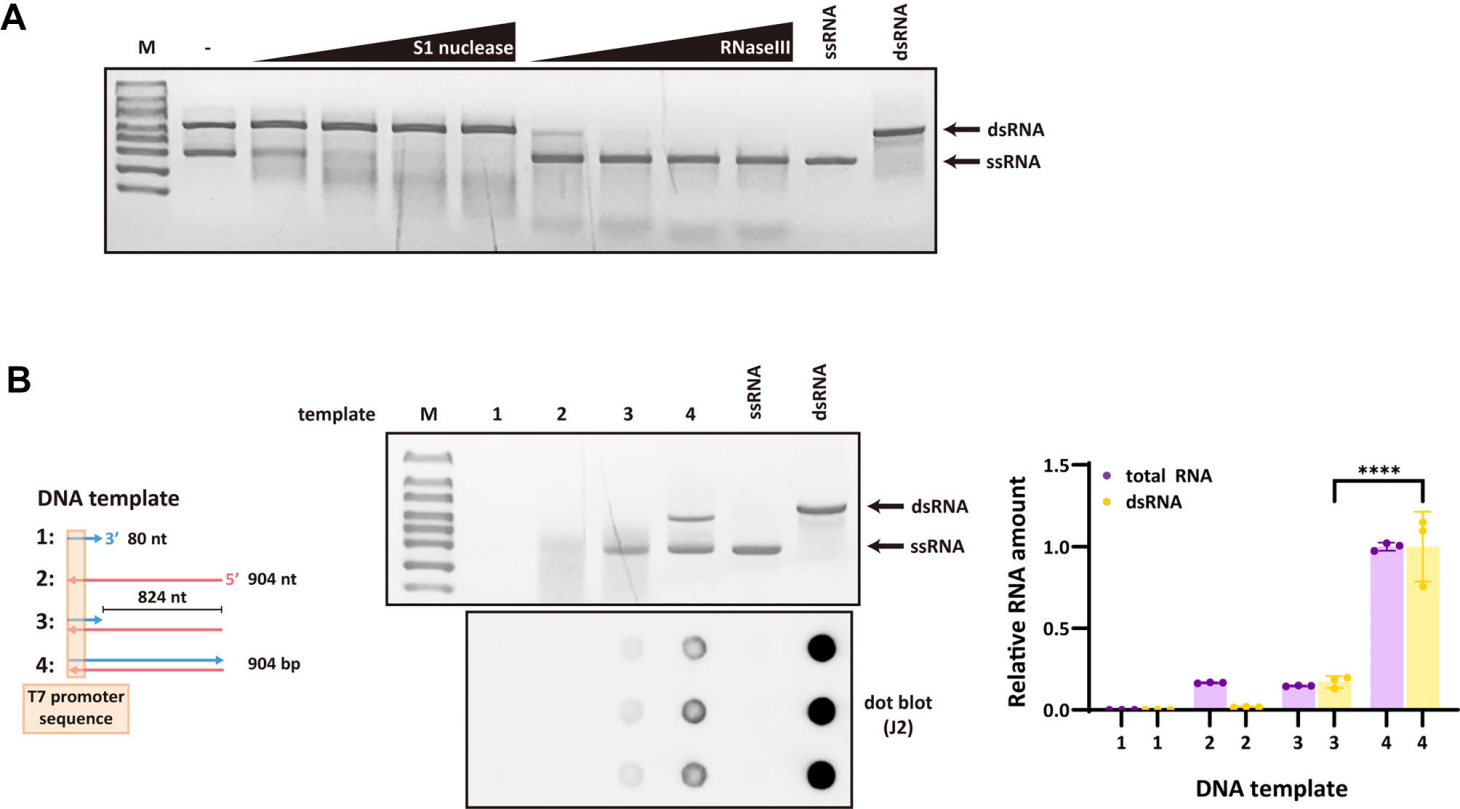
Promoter-independent antisense transcription contributes to dsRNA. (**A**) 1% agarose gel electrophoresis analysis of eGFP RNA synthesized by T7 RNA polymerase and treated with increasing concentrations of S1 nuclease (0.04, 0.08, 0.16, and 0.24 U) or RNase III (0.002, 0.004, 0.008, and 0.016 U). (**B**) 1% agarose gel and J2 antibody-based dot blot analysis of RNA transcribed from four DNA templates: (i) ssDNA containing only the T7 promoter region (80 nt), (ii) single-stranded template strand (904 nt), (iii) partially duplexed DNA with 80 bp encompassing the T7 promoter and a single-stranded downstream region, and (iv) fully duplexed DNA. For both analyses, 200 ng of each IVT RNA was loaded. As controls, 200 ng of ssRNA and 20 ng of dsRNA markers were included on the agarose gel, and 200 ng of ssRNA and 10 ng of dsRNA were spotted on the dot blot to confirm J2 antibody specificity. Total RNA was quantified using the RiboGreen assay. Values for total RNA and dsRNA were normalized to the yield obtained from the fully duplexed dsDNA template. Statistical comparisons were performed using one-way ANOVA with Šídák’s multiple comparisons test; ^****^P < .0001.

We first characterized the structure and origin of RNA products generated from conventional dsDNA templates (Fig. 1A). Native agarose gel electrophoresis revealed two distinct RNA bands corresponding to the positions of ssRNA and dsRNA markers. The upper bands in native agarose gels represent dsRNA formed by annealing between sense and antisense transcripts. Due to the strong intercalation of GelRed into duplex RNA, dsRNA bands appear more intense than ssRNA, even at lower molar input. Therefore, gel intensity alone does not reflect equal transcription levels between sense and antisense strands. To determine the identity of each band, we performed nuclease digestion assays. The upper band was eliminated by RNase III, as a dsRNA-specific endonuclease, but was not cleaved by S1 nuclease, which digests single-stranded nucleic acids. In contrast, the lower band was sensitive to S1 nuclease and resistant to RNase III. These results indicate that the upper band represents dsRNA, while the lower band corresponds to ssRNA.

To elucidate whether dsRNA formation results from promoter-independent antisense transcription, we employed a series of DNA templates with precisely defined duplex and single-stranded configurations (Fig. 1B). Template 1 consisted entirely of the non-template strand (80 nt) containing the T7 promoter sequence. Template 2 was derived from a full-length dsDNA template by selective removal of the non-template strand, yielding only the template strand (904 nt; Supplementary Fig. S1A). Template 3 featured a reconstituted T7 promoter region generated through annealing of template 1 to template 2 (Supplementary Fig. S1B). This design was employed to enable promoter-dependent transcription while preventing antisense transcription initiation, as the single-stranded downstream region eliminates the dsDNA terminus that serves as an initiation site for promoter-independent antisense transcription [8]. Template 4 served as a control, consisting of conventional dsDNA throughout its entire length.

Template 1 did not yield detectable RNA products, confirming that a single-stranded promoter was insufficient to initiate transcription. Unexpectedly, template 2 generated a heterogeneous smear of RNA products, indicating that T7 RNAP can initiate transcription in a promoter-independent manner at exposed 3′ ssDNA termini [27]. Template 3 generated both full-length transcripts and the smeared products observed with template 2, suggesting that unpaired downstream regions act as unintended substrates for abnormal initiation. Template 4 generated substantial quantities of dsRNA, as evidenced by the appearance of upper bands on native agarose gels and a strong signal in J2 antibody-based dot blot assays. Template 3 showed absence of these dsRNA bands, indicating substantially reduced dsRNA formation. Quantitative analysis of dot blot intensities revealed a ∼80% reduction in dsRNA with template 3 relative to template 4, while total RNA yield measured by RiboGreen decreased by ∼80%. These results indicate that dsRNA byproducts primarily originate from promoter-independent antisense transcription at the downstream dsDNA terminus.

### Template nicking suppresses dsRNA formation while maintaining RNA output

**Figure 2.**
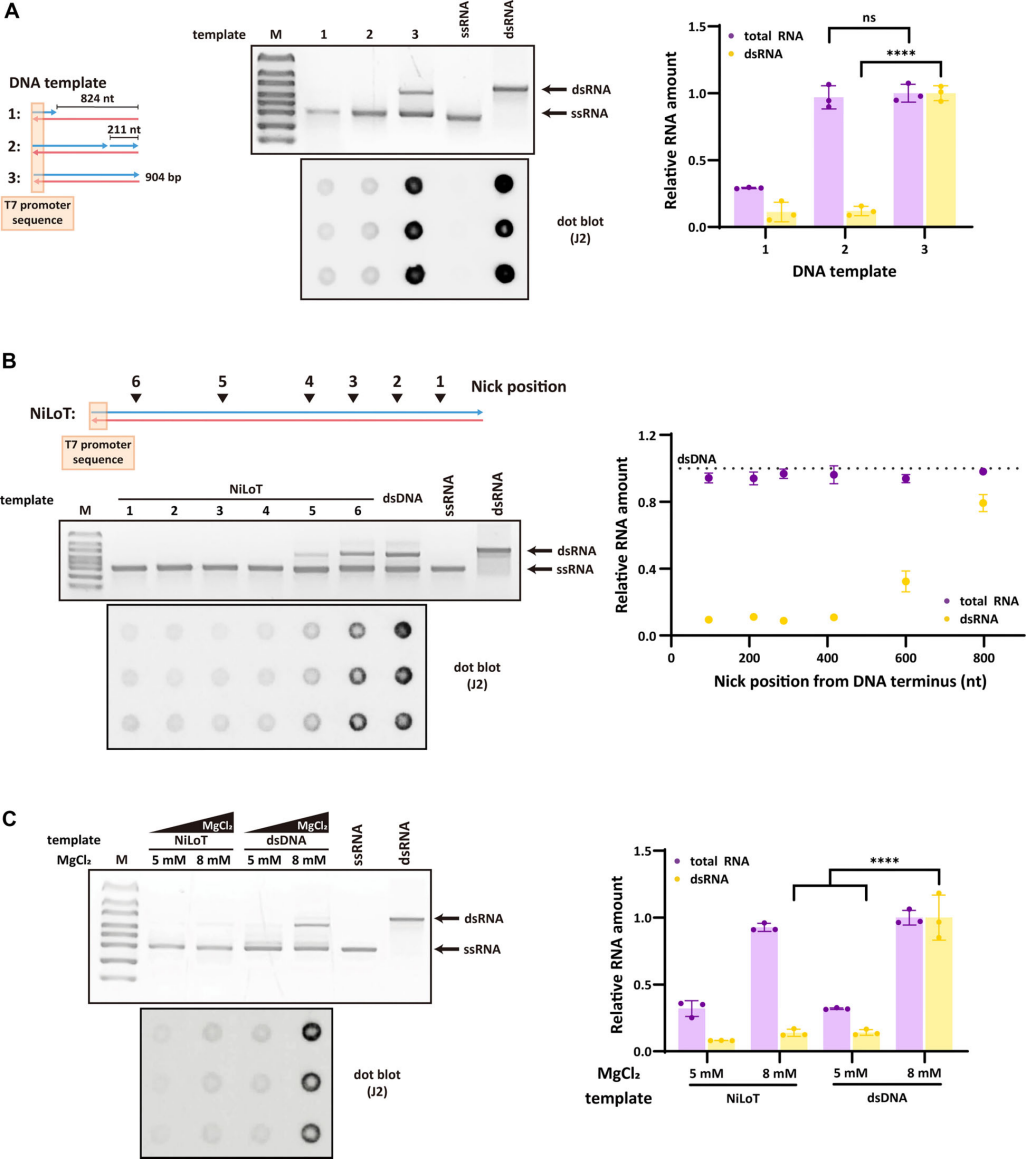
NiLoT reduces dsRNA without compromising RNA yield or integrity. (**A**) 1% agarose gel and J2 antibody-based dot blot analysis of RNA transcribed from three DNA templates: (i) partially duplexed DNA with 80 bp surrounding the T7 promoter and a single-stranded downstream region (no nick), (ii) nicked DNA (NiLoT) with a site-specific nick at 211 nt upstream of the 3′ end in the non-template strand, and (iii) fully duplexed DNA. 200 ng of each IVT RNA was analyzed. For reference, 200 ng of ssRNA and 20 ng of dsRNA markers were loaded on the gel, and 200 ng of ssRNA and 10 ng of dsRNA controls were included on the dot blot to validate antibody specificity. Total RNA was quantified using the RiboGreen assay. Values for total RNA and dsRNA were normalized to the yield obtained from the fully duplexed DNA template. Statistical comparisons were performed using one-way ANOVA with Šídák’s multiple comparisons test; ^****^P < .0001 and ns = not significant. (**B**) 1% agarose gel and J2 antibody-based dot blot analysis of RNA transcribed using NiLoT with nicks positioned at various distances (97, 211, 289, 417, 601, and 799 nt) from the 3′ end of the non-template strand (conditions 1–6). 200 ng of each IVT RNA was loaded for both analyses. For reference, 200 ng of ssRNA marker and 20 ng of dsRNA marker were loaded on the agarose gel. Total RNA was quantified using the RiboGreen assay and normalized to the yield obtained from the fully duplexed dsDNA template. (**C**) Comparison of NiLoT with MgCl₂-mediated dsRNA suppression. 1% agarose gel and J2 antibody-based dot blot analysis of RNA transcribed using standard dsDNA templates under two conditions: low MgCl₂ (5 mM) and NiLoT. For both analyses, 200 ng of each IVT RNA was loaded to allow direct comparison of RNA integrity and dsRNA content. For reference, 200 ng of ssRNA marker and 20 ng of dsRNA marker were loaded on the agarose gel, and 200 ng of ssRNA and 10 ng of dsRNA controls were included on the dot blot. Total RNA yield was independently quantified by RiboGreen assay, and bar graph values reflect total RNA recovered per IVT reaction. RNA and dsRNA levels were normalized to the yield obtained from the dsDNA template under 8 mM MgCl₂ conditions. Statistical comparisons were performed using one-way ANOVA with Šídák’s multiple comparisons test; ^****^P < .0001.

To evaluate whether introducing a nick into the non-template strand could suppress antisense transcription (Fig. 2A), we designed three DNA templates: partially ssDNA (template 1; identical to template 3 in Fig. 1B), nicked DNA (template 2, designated NiLoT), and fully dsDNA (template 3). Template 2 was generated by treating dsDNA with strand-specific nicking endonuclease Nt.Bpu10I, which introduced a site-specific SSB 211 nt from the 3′ DNA terminus in the non-template strand. Efficient nick formation was confirmed by S1 nuclease digestion, which selectively cleaves SSBs to yield fragmentation patterns (Supplementary Fig. S2).

NiLoT-based IVT significantly reduced dsRNA levels compared to the fully double-stranded control, as evidenced by decreased upper bands on native gels and reduced J2 antibody signals, without compromising the overall RNA yield. Notably, the extent of dsRNA reduction achieved with NiLoT was similar to that observed with template 1, which lacks a downstream double-stranded terminus. To further assess RNA integrity, we analyzed IVT products using the Agilent TapeStation system. NiLoT and fully duplexed templates both yielded RINe scores of 10.0, while the partially duplexed template gave a lower RINe of 9.2. The RINe (RNA Integrity Number equivalent) is a metric ranging from 1 (degraded RNA) to 10 (highly intact RNA), derived from RNA size distribution and degradation analysis. The electropherogram for the partial duplex product showed increased shorter fragments, and smear quantification (47–572 nt range) revealed ∼25% signal in the partial duplex template compared to ∼10% in both NiLoT and dsDNA templates (Supplementary Fig. S3A). These data confirm that NiLoT preserves RNA integrity and suppresses smeared transcript formation. Consistent with this mechanism, S1 nuclease digestion revealed that transcripts generated from NiLoT was ssRNA, whereas dsDNA-derived transcripts retained significant dsRNA (Supplementary Fig. S3B). These observations support a mechanistic model in which a site-specific nick impedes promoter-independent antisense transcription initiation at the DNA terminus, thereby reducing the formation of antisense RNA products. To determine whether the reduction in dsRNA levels was directly attributable to NiLoT, we conducted IVT reactions using mixtures of dsDNA and NiLoT led to a gradual decrease in dsRNA levels without affecting total RNA yield (Supplementary Fig. S4). Quantification showed a linear inverse relationship between NiLoT content and dsRNA accumulation.

To investigate the impact of nick position on antisense suppression, we tested templates with nicks at defined distances from the DNA terminus (Fig. 2B). Cas9 D10A nickase was used in combination with sgRNAs targeting sites located 97, 289, 417, 601, 799 nt from the terminus (Supplementary Fig. S2B−C). In parallel, the 211 nt condition was included using the previously described NiLoT generated by Nt.Bpu10I. Site-specific nick formation was confirmed by S1 nuclease digestion analysis (Supplementary Fig. S2A). dsRNA formation was markedly reduced when the nick was positioned within ∼400 nt of the 3′ DNA terminus in the non-template strand. Beyond this threshold, dsRNA levels progressively increased as the nick site moved further upstream. Notably, total RNA yield remained comparable across all conditions regardless of nick position. These results suggest that effective inhibition of promoter-independent antisense transcription requires the nick to be positioned within a certain distance from the 3′ DNA terminus in the non-template strand.

A previous study demonstrated that lowering MgCl_2_ concentration is effective in reducing promoter-independent antisense transcription [8]. To compare this strategy with NiLoT, we conducted IVT reactions using either standard or reduced MgCl_2_ conditions (Fig. 2C). Decreasing MgCl_2_ from 8 mM to 5 mM led to a marked reduction in dsRNA formation, comparable to that achieved using NiLoT. In contrast to NiLoT, which preserved total RNA yield, the reduced MgCl_2_ condition significantly decreased RNA output [6, 20]. These findings establish NiLoT as an effective template-based strategy for minimizing dsRNA formation without altering the chemistry of standard IVT reactions. We next evaluated the compatibility of NiLoT with urea, a chaotropic reagent reported to suppress RNA self-priming [19] (Supplementary Fig. S5). Addition of 0.2 − 0.8 M urea during IVT with dsDNA templates reduced dsRNA signals on dot blots but did not eliminate full-length dsRNA bands. In contrast, NiLoT alone markedly diminished full-length dsRNA, and combining NiLoT with 0.2 − 0.8 M urea further decreased dsRNA levels, indicating a synergistic effect between NiLoT and chaotropic conditions. At higher urea concentration, total RNA yield declines moderately, and at 1.0 M urea, RNA synthesis was severely inhibited, resulting in minimal product formation. Together, these results demonstrate that NiLoT is broadly compatible with existing dsRNA-reduction strategies and enables further suppression of dsRNA contaminants.

### R-loop formation drives strand displacement in NiLoT templates

**Figure 3.**
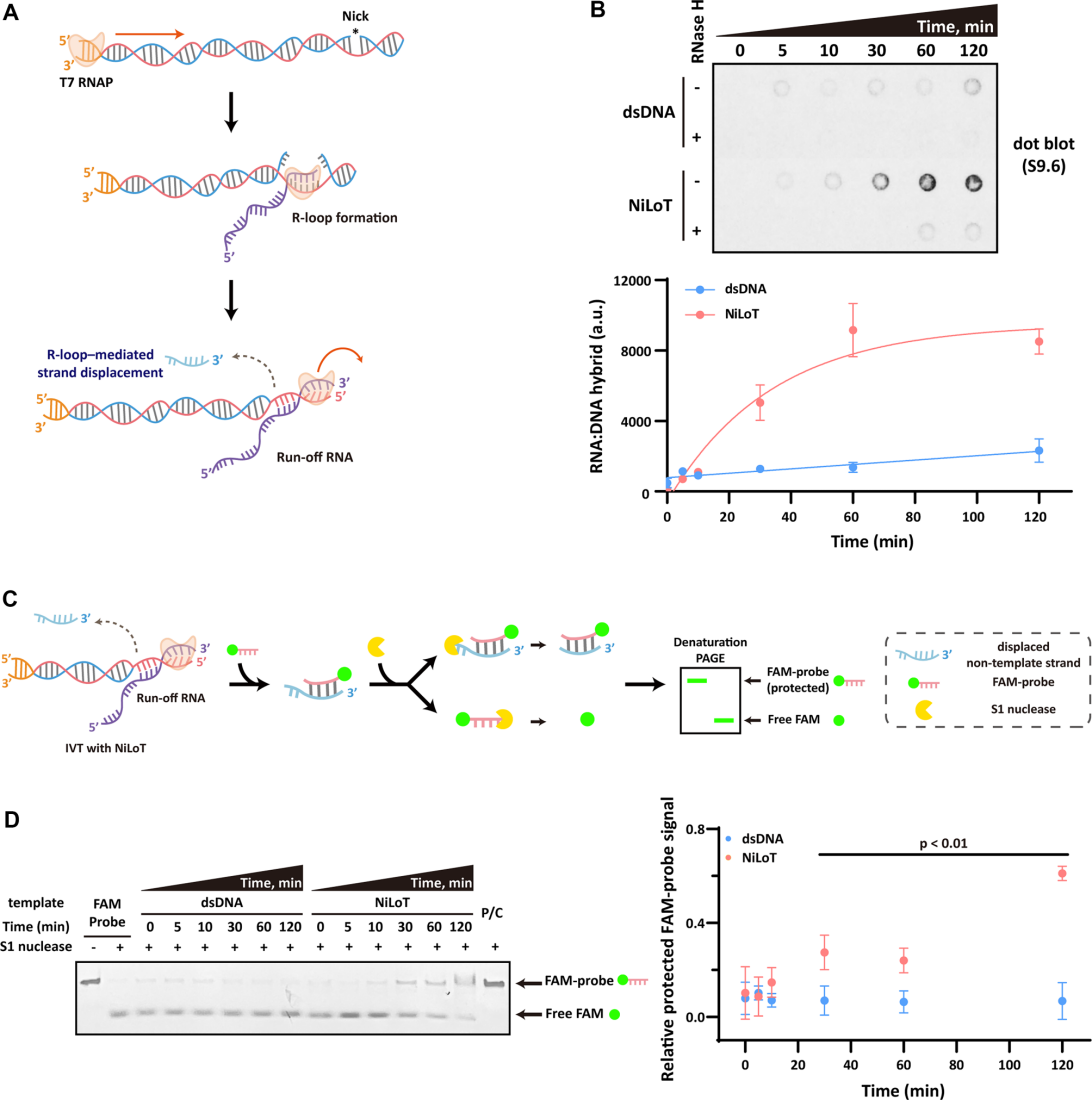
NiLoT induces strand displacement via R-loop formation. (**A**) Schematic illustration of the proposed mechanism: T7 RNAP transcription induces R-loop formation at the nick site, resulting in displacement of the non-template strand and inhibition of antisense transcription initiation. (**B**) Dot blot analysis using the S9.6 antibody to detect RNA:DNA hybrids over a time-course IVT reaction using either dsDNA or NiLoT. Nonlinear regression was performed using a one-phase association model (least-squares method, GraphPad Prism version 8.0.2). The fitting equation was $Y = \ {{Y}_0} + ( {\mathrm{Plateau} - \ {{Y}_0}} ) \times ( {1 - {{e}^{ - kx}}} )$. Detailed fitting parameters and 95% confidence intervals are provided in Supplementary Table S3. (**C**) Schematic of the S1 nuclease protection assay designed to detect strand displacement by hybridization of a FAM-labeled probe to the displaced non-template strand. (**D**) 10% denaturing PAGE analysis of time-course IVT reactions using either dsDNA or NiLoT templates, following FAM-probe hybridization and S1 digestion. P/C: Positive control, generated by pre-annealing the 30 nt FAM-labeled probe with a fully complementary 30 nt oligonucleotide before S1 nuclease digestion. Band intensities of protected FAM-probe were quantified and normalized to the positive control. Statistical comparisons were performed using one-way ANOVA with Šídák’s multiple comparisons test.

When a nick is introduced into the non-template strand, the nascent RNA can more effectively form a RNA:DNA hybrid (R-loop) during transcription [21]. This R-loop formation facilitates strand displacement of the downstream non-template region [27]. Based on this, we hypothesized that nicking the non-template strand promotes R-loop-mediated strand displacement, thereby preventing promoter-independent antisense transcription from the exposed DNA terminus (Fig. 3A).

To test this model, we monitored R-loop formation over time by performing S9.6 antibody dot blot analysis of IVT reactions using either dsDNA or NiLoT (Fig. 3B). While dsDNA templates showed only basal levels of RNA:DNA hybrid formation, NiLoT displayed a time-dependent increase in R-loop signal. This signal was abolished upon RNase H treatment, confirming its specificity for RNA:DNA hybrids. These findings suggest that NiLoT facilitates RNA:DNA hybrid formation by increasing template strand availability.

After confirming R-loop formation in NiLoT, we developed an S1 nuclease protection assay to assess whether this promoted strand displacement (Fig. 3C) [29]. Following IVT, total nucleic acids were purified and incubated with a 30 nt FAM-labeled DNA probe complementary to the 3′ end of the non-template strand (Supplementary Table S1). FAM-probe signals were negligible in reactions using dsDNA template. Unlike dsDNA template, NiLoT exhibited detectable probe protection beginning at 10 min, with statistically significant increases observed at 30, 60, and 120 min (*P* < .01; Fig. 3D). These signals indicated that strand displacement exposed the non-template strand, allowing probe annealing and protection from S1 nuclease digestion. Together, these findings indicate that NiLoT facilitates progressive strand displacement over time.

To examine whether the nick position-dependent suppression of dsRNA by NiLoT (Fig. 2B) is reflected in R-loop formation and strand displacement efficiency, we first performed S9.6 antibody-based dot blot analysis (Supplementary Fig. S6A). The R-loop signal increased as the nick was positioned farther from the terminus, reaching a maximum at ∼400 nt. Beyond this point, the signal plateaued and slightly decreased, indicating that efficient R-loop formation becomes progressively limited once the displaced region exceeds this length, resulting in incomplete strand displacement. Consist with this interpretation, we conducted S1 nuclease protection assays (Supplementary Fig. S6B). Probe signals declined when the nick was placed beyond ∼400 nt from the terminus, correlating with restored dsRNA formation. These findings define a functional threshold of ∼400 nt for stable R-loop-mediated strand displacement.

### NiLoT maintains dsRNA suppression under varied IVT conditions

**Figure 4.**
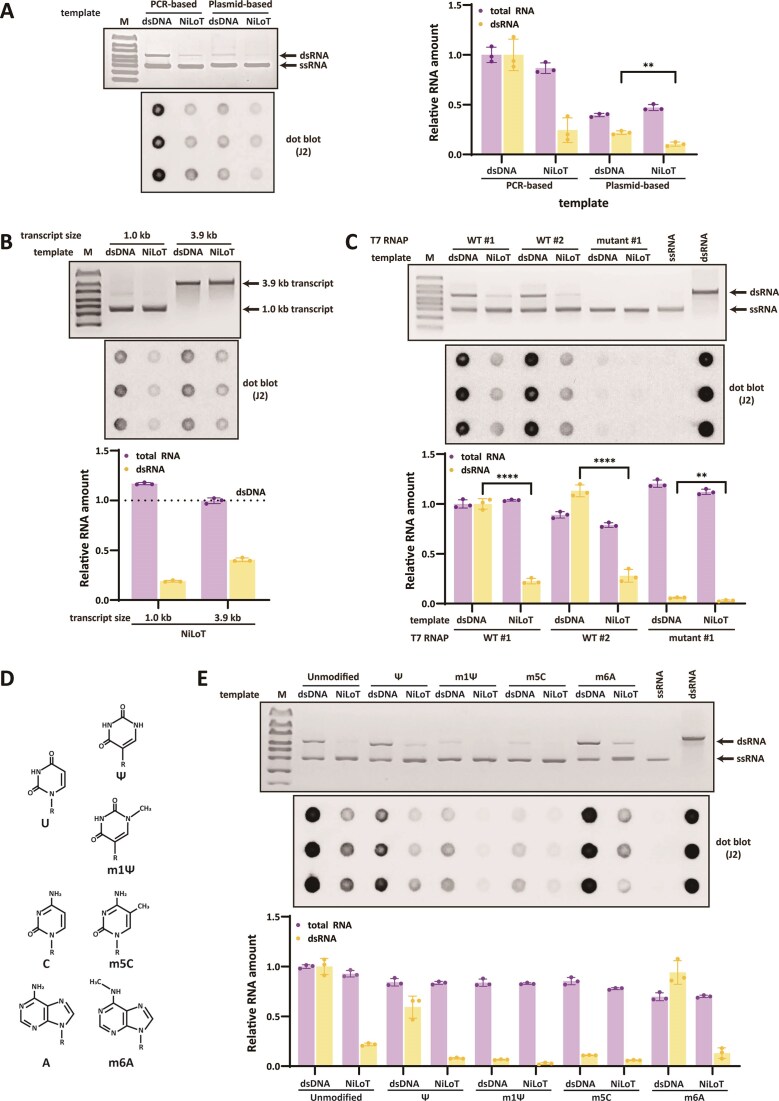
NiLoT suppresses dsRNA across different template formats, transcript sizes, T7 RNAP variants, and modified NTPs. (**A**) 1% agarose gel and J2 antibody-based dot blot analysis of RNA transcribed using either PCR-amplified or linearized plasmid DNA templates, prepared as dsDNA or NiLoT. 200 ng of each IVT RNA was loaded for both analyses. For reference, 200 ng of ssRNA marker and 20 ng of dsRNA marker were loaded on the agarose gel. Total RNA was quantified using the RiboGreen assay. Values for total RNA and dsRNA were normalized to the yield obtained from the PCR-based dsDNA template with unmodified NTPs. Statistical comparisons were performed using two-tailed, unpaired *t*-test; **P < .01. (**B**) Agarose gel electrophoresis and J2 dot blot analysis of IVT products from 1.0 kb and 3.9 kb templates. RNAs were synthesized using either dsDNA or NiLoT templates encoding 1.0 kb (eGFP) or 3.9 kb (SARS-CoV-2 spike) transcripts. For each condition, 200 ng of IVT RNA was analyzed by 1% agarose gel electrophoresis and by dot blot using the J2 antibody. For reference, 200 ng of ssRNA and 20 ng of dsRNA were loaded on the gel as controls. Total RNA concentrations were measured using the RiboGreen assay, and dsRNA levels were quantified from dot blots. All values were normalized to the total RNA and dsRNA yield obtained from the corresponding dsDNA-derived transcripts. (**C**) Representative 1% agarose gel and J2 antibody-based dot blot analyses of IVT RNA transcribed from either conventional dsDNA or NiLoT templates using three T7 RNA polymerases: WT #1 (vendor 1, wild-type), WT #2 (vendor 2, wild-type), and Mutant #1 (vendor 1, engineered low-dsRNA variant). For each condition, 200 ng of RNA was loaded per assay. Reference lanes include 200 ng ssRNA and 20 ng dsRNA markers (gel), and 200 ng ssRNA and 10 ng dsRNA controls (dot blot). Total RNA was quantified using the RiboGreen assay. RNA yield and dsRNA content were normalized to the output from the dsDNA template transcribed with WT #1. Statistical comparisons were performed using a two-tailed, unpaired *t*-test; **P < .01 and ^****^P < .0001. (**D**) Chemical structures and abbreviations of modified nucleotides used for IVT: Ψ (pseudouridine), m^1^Ψ (N1-methylpseudouridine), m^5^C (5-methylcytidine), and m^6^A (N6-methyladenosine). (**E**) 1% agarose gel and J2 antibody–based dot blot analysis of eGFP mRNA transcribed using either dsDNA or NiLoT templates, with or without modified NTPs shown in panel (**D**). 200 ng of IVT mRNA was loaded per lane/spot. For reference, 200 ng of ssRNA marker and 20 ng of dsRNA marker were loaded on the agarose gel, and 200 ng of ssRNA and 10 ng of dsRNA controls were included on the dot blot. The J2 blot confirms that NiLoT-derived mRNAs contain markedly lower levels of dsRNA contaminants than dsDNA-derived counterparts. Total RNA was quantified using the RiboGreen assay. Values for total RNA and dsRNA were normalized to the yield obtained from the dsDNA template with unmodified NTPs.

To evaluate the generalizability of NiLoT-mediated dsRNA suppression, we tested its efficacy across commonly used mRNA template formats (Fig. 4A). We compared PCR-amplified linear DNA with linearized plasmid DNA containing terminal poly (A) sequence and 5′ overhangs [30]. Plasmid-based templates consistently yielded lower total RNA than PCR-derived templates, regardless of nicking. However, within each template format, NiLoT produced equivalent or greater RNA output relative to the corresponding double-stranded control. Importantly, NiLoT reduced dsRNA levels across both template types. Plasmid-based templates generated less dsRNA than PCR-derived ones, likely due to terminal structural features [8, 11, 27, 31]. NiLoT further suppressed dsRNA formation, providing an additive effect. These results show that NiLoT reliably reduces dsRNA formation while maintaining RNA yield across diverse template configurations.

To evaluate the applicability of NiLoT to longer and more structured transcripts, we tested a 3.9 kb SARS-CoV-2 spike protein mRNA, which represents a clinically relevant therapeutic length (Fig. 4B). NiLoT suppressed dsRNA formation in this large transcript as effectively as in the 1.0 kb eGFP model. Importantly, the overall RNA yield was not reduced. These results demonstrate that NiLoT remains effective for long, structured mRNAs representative of therapeutic applications. To assess the generality of NiLoT across different transcription enzymes, we examined its performance using three T7 RNAPs: two wild-type enzymes and one engineered low-dsRNA variant (Fig. 4C). NiLoT consistently reduced dsRNA levels across all polymerases, while maintaining comparable total RNA yields. The engineered T7 RNAP generated less dsRNA than wild-type enzymes. NiLoT combined with the engineered T7 RNAP further decreased dsRNA formation. These results demonstrate that NiLoT is compatible with both wild-type and engineered T7 RNAPs and offer additional dsRNA reduction when combined with low-dsRNA polymerases.

To further evaluate whether NiLoT maintains its dsRNA-suppressive capacity in the context of modified nucleotides, we conducted IVT reactions using either dsDNA or NiLoT templates with unmodified NTPs or complete substitution of uridine, cytidine, or adenosine with Ψ, m¹Ψ, m^5^C, or m^6^A, respectively (Fig. 4D and E). When standard dsDNA templates were used, incorporation of Ψ, m¹Ψ, and m^5^C moderately reduced dsRNA formation, whereas m^6^A failed to exert such an effect, consistent with prior observations [8]. In contrast, NiLoT consistently suppressed dsRNA levels across all NTP compositions, regardless of the nucleotide modification used. Notably, RNA yield remained unchanged across all NiLoT conditions, indicating that the improved dsRNA purity did not come at the cost of transcriptional efficiency. These findings demonstrate that NiLoT’s dsRNA-reducing effect is robust and synergistic with chemical modifications, underscoring its compatibility with standard mRNA.

### NiLoT improves mRNA function and reduces immunogenicity

To evaluate the functional consequences of dsRNA reduction on protein expression and innate immune activation, we first examined the polyadenylation efficiency of eGFP mRNAs synthesized from either dsDNA or NiLoT templates using various modified NTPs (Supplementary Fig. S7). Enzymatic polyadenylation caused a distinct mobility shift in NiLoT-derived mRNAs, indicating that these transcripts that harbor modified nucleotides remained competent for 3′ tailing.

**Figure 5.**
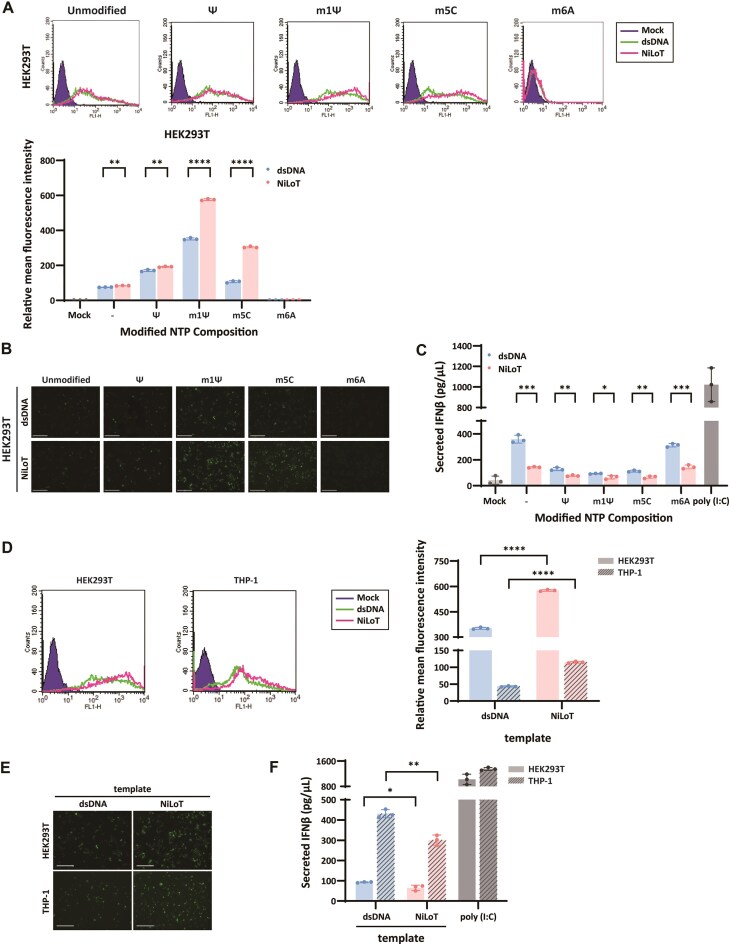
NiLoT-derived mRNA enhances translation and reduces immune activation. (**A**) Flow cytometry analysis of eGFP expression in HEK293T cells transfected with eGFP mRNA synthesized using either dsDNA or NiLoT, as used in Fig. 4E. Fluorescence intensity was measured at 18 h post-transfection. Cells treated with Lipofectamine^TM^ 3000 alone served as mock controls. Mean fluorescence intensity was normalized to the mock condition. Statistical comparisons were performed using two-tailed, unpaired *t*-test; **P < .01 and ^****^P < .0001. (**B**) Fluorescence microscopy images showing eGFP expression in cells transfected with NiLoT- or dsDNA-derived eGFP mRNA. (**C**) Quantification of IFN-β secretion by ELISA in HEK293T cells transfected with NiLoT- or dsDNA-based eGFP mRNA. Cells treated with Lipofectamine^TM^ 3000 alone served as mock controls, and cells treated with poly(I:C) served as positive controls for immune activation. Statistical comparisons were performed using two-tailed, unpaired *t*-test; *P < .05, **P < .01, and ***P < .001. (**D**) Flow cytometry analysis of eGFP expression in HEK293T and THP-1 cells transfected with m^1^Ψ-modified eGFP mRNA synthesized using either dsDNA or NiLoT. Fluorescence intensity was measured at 18 h post-transfection. Cells treated with Lipofectamine 3000 alone served as mock controls. Mean fluorescence intensity was normalized to the mock condition. Statistical comparisons were performed using two-tailed, unpaired *t*-test; ^****^P < .0001. (**E**) Fluorescence microscopy images showing eGFP expression in HEK293T and THP-1 cells transfected with NiLoT- or dsDNA-derived eGFP mRNA. (**F**) Quantification of IFN-β secretion by ELISA in HEK293T and THP-1 cells transfected with m^1^Ψ-modified eGFP mRNA synthesized from either NiLoT or dsDNA templates. Cells treated with poly(I:C) were included as positive controls for innate immune activation. ELISA measurements were performed 24 h post-transfection. Statistical comparisons were performed using a two-tailed, unpaired *t*-test; *P < .05 and **P < .01.

Next, we assessed translational output in HEK293T cells by transfecting polyadenylated mRNAs and measuring eGFP expression via flow cytometry (Fig. 5A). Across all tested nucleotide conditions, such as unmodified, Ψ, m¹Ψ, and m^5^C, NiLoT-derived mRNAs consistently yielded higher eGFP fluorescence compared to their dsDNA-derived counterparts. The enhancement was most pronounced with m¹Ψ incorporation, suggesting a potential synergistic effect between the NiLoT template and specific base modifications. In contrast, m^6^A-modified mRNAs failed to produce detectable fluorescence regardless of template origin. Fluorescence microscopy corroborated these findings, revealing more robust and widespread eGFP signals in cells transfected with NiLoT-derived mRNAs (Fig. 5B). Importantly, IFN-β secretion assays demonstrated that NiLoT-derived mRNAs induced significantly lower innate immune responses across all NTP conditions, relative to dsDNA-derived transcripts (Fig. 5C). These results confirm that NiLoT reduces immunogenicity by minimizing dsRNA contaminants, while simultaneously preserving compatibility with commonly used nucleotide modifications.

To assess whether the benefits of NiLoT-based transcription extend to various cell types, we transfected both HEK293T cells and immune-relevant THP-1 monocytes with m¹Ψ-modified eGFP mRNAs synthesized from either standard dsDNA or NiLoT templates (Fig. 5D−F). Flow cytometry analysis revealed that NiLoT-derived mRNAs consistently produced higher eGFP fluorescence than dsDNA-derived counterparts in both cell types (Fig. 5D). Fluorescence microscopy further supported these findings, showing more intense and widespread eGFP expression in cells transfected with NiLoT-derived mRNA (Fig. 5E). To evaluate innate immune activation, we measured IFN-β secretion in the cell culture supernatants using ELISA. As shown in Fig. 5F, NiLoT-derived mRNAs elicited significantly lower IFN-β production than those transcribed from dsDNA templates in both HEK293T and THP-1 cells. These results confirm that NiLoT not only enhances protein expression from IVT mRNA but also minimizes immunogenicity, validating its utility for immunologically sensitive applications such as therapeutic mRNA development.

To investigate the nature of residual nucleic acid contaminants and their immunostimulatory potential, we performed S9.6 antibody-based dot blot analysis alongside functional assays in THP-1 cells, which possess intact cGAS–STING and TLR3 signalling pathways [32]. Although NiLoT-derived IVT products initially showed higher RNA:DNA hybrid content compared to dsDNA-derived transcripts, these hybrids were efficiently eliminated following standard DNase I treatment, resulting in comparable hybrid levels between the two template types (Supplementary Fig. S8A).

To delineate the contribution of distinct nucleic acid species to immune activation, we transfected THP-1 cells with IVT RNAs derived from dsDNA or NiLoT templates subjected to selective nuclease treatments: (i) untreated, (ii) RNase H only, (iii) DNase I only, (iv) DNase I and RNase H, and (v) DNase I and RNase III (Supplementary Fig. S8B). In dsDNA-derived samples, IFN-β secretion decreased progressively following RNase H and DNase I digestion, with the most pronounced drop occurring after DNase I treatment, implicating residual DNA as the primary immune activator—consistent with cGAS–STING pathway activation. Additional RNase III treatment further reduced IFN-β levels, confirming the immunogenic contribution of dsRNA contaminants. In contrast, NiLoT-derived RNA consistently elicited lower IFN-β responses under all conditions. Notably, after DNase I treatment, neither RNase H nor RNase III conferred further reductions in IFN-β secretion, indicating that RNA:DNA hybrids and dsRNA are effectively minimized in NiLoT preparations. These results demonstrate that while residual DNA may transiently stimulate innate immunity, it is efficiently removed by standard DNase I treatment. After DNA elimination, dsRNA remains the dominant immunostimulatory contaminant in conventional IVT, whereas NiLoT intrinsically suppresses dsRNA generation, thereby minimizing IFN-β induction without the need for additional enzymatic purification.

## Discussion

T7 RNAP-based IVT generates dsRNA byproducts that activate innate immune responses and reduce translation efficiency [6, 7]. We identified promoter-independent antisense transcription from exposed duplex DNA ends as the primary dsRNA source (Fig. 1). NiLoT, a template design strategy that introduces site-specific nicks in the non-template strand, effectively suppresses this process (Fig. 2). NiLoT introduces SSB within ∼400 nt of the 3′ DNA terminus. This facilitates R-loop formation during sense transcription, displacing the downstream non-template strand and preventing antisense initiation at exposed termini (Fig. 3). The half-time of R-loop formation was ~23 min that was calculated from the exponential rate (k) of R-loop formation kinetics with NiLoT (Supplementary Table S3), while strand displacement became statistically significant at 30 min, suggesting that strand displacement is driven by prior R-loop accumulation.

When the nick was placed beyond ∼400 nt, both hybrid and protection signals decreased, and dsRNA suppression was lost, defining a positional limit for stable R-loop propagation and efficient antisense inhibition (Supplementary Fig. S6). R-loop formation was most efficient when the nick was positioned within ∼400 nucleotides of the 3′ end of the non-template strand, with hybrid signal intensity plateauing or slightly declining beyond this distance. This suggests that nick-induced strand displacement operates within a finite propagation range. A likely explanation is that the displaced DNA fragment, when excessively long, begins to compete with the nascent RNA for access to the template strand, thereby destabilizing the R-loop structure and limiting its extension. This mechanistic threshold corresponds with the observed decline in NiLoT efficacy at greater distances and highlights the importance of optimal nick positioning for maximal hybrid induction and dsRNA suppression. Together, these findings reveal that R-loop-mediated strand displacement underlies NiLoT-mediated suppression of promoter-independent antisense transcription. A strategically positioned SSB can thus direct the polarity of T7 RNAP transcription.

NiLoT reduced dsRNA levels comparable to Mg^2+^ depletion while preserving RNA yield (Fig. 2C). Unlike ionic or buffer optimization strategies, NiLoT suppresses dsRNA formation purely through template design, without altering standard IVT conditions. NiLoT was compatible with chaotropic reagents such as urea, known to suppress RNA self-priming [19]. Combining NiLoT with 0.2−0.8 M urea further reduced dsRNA levels in a synergistic manner (Supplementary Fig. S5), confirming its orthogonality with other reduction strategies.

NiLoT was effective in plasmid-based templates containing 5′ overhangs and poly(A) tails (Fig. 4A). While these features partially suppressed antisense transcription, dsRNA remained detectable [8]. Nicking provided additional reduction, demonstrating an additive effect. Lower RNA yields from plasmid templates compared to PCR-derived templates may reflect structural differences after restriction digestion [33, 34]. Importantly, NiLoT effectively suppressed dsRNA formation in a 3.9 kb SARS-CoV-2 spike mRNA transcript (Fig. 4B), demonstrating its robustness for long, structured, and clinically relevant RNAs. NiLoT was also compatible with multiple T7 RNAPs, including wild-type and engineered low-dsRNA variants (Fig. 4C). NiLoT also retained efficacy across several nucleotide modifications. Ψ, m^1^Ψ, m^5^C individually reduced dsRNA levels [8], and combining them with NiLoT provided further suppression (Fig. 4D and E).

Functionally, NiLoT-derived mRNAs exhibited higher protein expression and lower innate immune activation than dsDNA-derived mRNAs in both HEK293T and THP-1 cells (Fig. 5D−F). In HEK293T cells, NiLoT-derived mRNAs enhanced eGFP expression regardless of NTP modification (Fig. 5A and B). The strongest effect occurred with m^1^Ψ-mRNAs, suggesting synergistic benefits between NiLoT and specific nucleotide modifications [35]. In contrast, m^6^A-modified mRNAs failed to produce detectable eGFP expression. This likely reflects excessive or non-physiological incorporation of m^6^A, which can impair translation through multiple mechanisms [36, 37]. For instance, aberrant enrichment of m^6^A may disrupt ribosome scanning or promote binding of inhibitory proteins. Recent study has shown that excessive m^6^A modification facilitates mRNA sequestration into P-bodies and represses translation through IGF2BP3-mediated regulation [38]. Such mechanisms may account for the strongly reduced expression observed in our study.

A potential concern is the presence of RNA:DNA hybrids formed during R-loop generation. These structures have been reported to activate innate immune sensors such as cGAS [32] and TLR9 [39], potentially inducing type I IFN responses. However, these hybrids are typically template-bound and transient during IVT. Such hybrids are effectively removed by the standard DNase I treatment routinely employed in RNA manufacturing workflows (Supplementary Fig. S8A). Consistently, additional RNase H or RNase III digestion of NiLoT-derived RNA did not further reduce IFN-β secretion in THP-1 cells (Supplementary Fig. S8B). This confirms that RNA:DNA hybrids play a minimal role in immune activation after DNase I treatment. Instead, dsRNA remains the principal immunostimulatory contaminant in conventional IVT preparations.

From a manufacturing perspective, NiLoT introduces a simple yet robust modification to standard dsDNA templates, enabling strand displacement without requiring complex synthesis steps or novel reagents. We observed that nicked templates remained stable for at least four months when stored at –20°C, with no measurable decline in transcriptional yield or fidelity. Importantly, nicking efficiency and template integrity can be reliably assessed using standard methods such as S1 nuclease digestion, denaturing PAGE, or NickSeq-based mapping [40], supporting quality control in large-scale production workflows.

In this study, NiLoT was implemented using both sequence-specific nicking endonucleases and the programmable nickase Cas9 D10A, enabling precise introduction of SSBs in the non-template DNA strand. While our data show that effective strand displacement requires the nicking site to be within ∼400 nt of the 3′ terminus, this positional constraint can be overcome through rational template design. Additionally, strategic placement of the nicking site within non-coding regions such as the 3′ untranslated region (UTR) can minimize any potential impact on protein-coding sequences, further enhancing manufacturing robustness. Specifically, pre-engineered 3′ UTR modules incorporating validated nicking motifs may be appended downstream of the coding region, thereby ensuring broad compatibility across diverse mRNA sequences without altering the transcript’s functional domains. Such modular UTR strategies, together with PAM-targeted nickases like Cas9 D10A, offer flexible and scalable avenues for applying NiLoT to a wide range of therapeutic mRNA constructs. Furthermore, NiLoT is compatible with other dsRNA-reducing strategies, including 5′ overhang templates, modified NTPs, engineered T7 RNAP, and chaotropic reagents, which enable synergistic suppression of dsRNA formation. While NiLoT proved effective across diverse conditions, further studies are warranted to evaluate the impact of multiple nicks or varied spacing.

To enhance translational relevance and enable practical benchmarking, we quantified absolute RNA yields for all tested IVT conditions, including dsDNA, NiLoT, partially duplexed templates, reduced MgCl₂ concentrations, and multiple RNA polymerase variants. These results, summarized in Supplementary Table S2, demonstrate that NiLoT consistently achieves RNA outputs comparable to conventional dsDNA templates while markedly reducing dsRNA levels. Importantly, this robust performance was maintained across diverse template architectures and enzymatic systems, highlighting the manufacturing compatibility of NiLoT.

In summary, NiLoT suppresses promoter-independent antisense transcription and reduces dsRNA formation without compromising RNA yield. It functions across multiple template types, nucleotide modifications, and T7 RNAP variants while improving protein expression and immune tolerance. As synthetic mRNA advances toward broader clinical use, NiLoT provides a simple and scalable solution to enhance IVT product quality and therapeutic consistency.

## Supplementary Material

gkaf1536_Supplemental_File

## Data Availability

No additional data were generated for this study.
