# Supplementary material for: Template nicking suppresses promoter-independent antisense transcription in IVT via R-loop-mediated strand displacement
Source: Nucleic Acids Res. 2026 Jan 15;54(2):gkaf1536. doi: 10.1093/nar/gkaf1536 (PMC12805894; doi:10.1093/nar/gkaf1536)
Supplement: gkaf1536_Supplemental_File [file gkaf1536_supplemental_file.docx]

**Supplementary Table S1.** Sequences of DNA oligonucleotides in this study.

| DNA | 5’ Modification | Sequence (5’-3’) |
| --- | --- | --- |
| PCR forward primer | Phosphorylation | TAA TAC GAC TCA CTA TAA GGA A |
| PCR reverse primer | - | GCC GCC CAC TCA GAC TTT AT |
| non-template strand containing the T7 promoter | - | TAA TAC GAC TCA CTA TAA GGA ATA AAC TAG TAT TCT TCT GGT CCC CAC AGA CTC AGA GAG AAC CCG CCA CCA TGG TGA GC |
| Target-specific oligo  (97 nt) | - | TTC TAA TAC GAC TCA CTA TAG GCA AGA AGC TAG GCC ACC GGT TTT AGA GCT AGA |
| Target-specific oligo  (289 nt) | - | TTC TAA TAC GAC TCA CTA TAG GTG TTC TGC TGG TAG TGG TGT TTT AGA GCT AGA |
| Target-specific oligo  (417 nt) | - | TTC TAA TAC GAC TCA CTA TAG TGT ACT CCA GCT TGT GCC CCG TTT TAG AGC TAG A |
| Target-specific oligo  (601 nt) | - | TTC TAA TAC GAC TCA CTA TAG TCG TGC TGC TTC ATG TGG TGT TTT AGA GCT AGA |
| Target-specific oligo  (799 nt) | - | TTC TAA TAC GAC TCA CTA TAG ACC AGG ATG GGC ACC ACC CGT TTT AGA GCT AGA |
| Strand displacement  capture probe | 6-FAM | GCC GCC CAC TCA GAC TTT ATT CAA AGA CCA |
| Strand displacement  positive control oligo | - | TGG TCT TTG AAT AAA GTC TGA GTG GGC GGC |

**Supplementary Table S2**. Absolute RNA yields measured by RiboGreen assay across all IVT conditions described in the main and supplementary figures. Data are presented as mean ± standard deviation (SD) from *n* = 3 independent biological replicates for each condition.

| Figure | Reaction condition | | RNA yield (μg) |
| --- | --- | --- | --- |
| Figure 1B | Template 1 | | Not detectable |
|  | Template 2 | | 4.51 ± 0.09 |
|  | Template 3 | | 4.00 ± 0.08 |
|  | Template 4 | | 27.19 ± 0.68 |
| Figure 2A | Template 1 | | 7.93 ± 0.13 |
|  | Template 2 | | 26.40 ± 2.34 |
|  | Template 3 | | 27.23 ± 1.80 |
| Supplementary  Figure S4 | dsDNA/NiLoT | 2.0/0.0 | 27.19 ± 0.39 |
|  |  | 1.5/0.5 | 28.04 ±1.66 |
|  |  | 1.0/1.0 | 28.51 ± 1.11 |
|  |  | 0.5/1.5 | 23.66 ± 2.35 |
|  |  | 0.0/2.0 | 25.60 ± 1.36 |
| Figure 2B | dsDNA | | 28.61 ± 0.73 |
|  | Nick position  (NiLoT) | 97 | 26.93 ± 0.82 |
|  |  | 211 | 27.30 ± 1.09 |
|  |  | 289 | 27.61 ±0.82 |
|  |  | 417 | 27.18 ± 1.52 |
|  |  | 601 | 27.45 ± 0.72 |
|  |  | 799 | 28.33 ± 0.61 |
| Figure 2C | NiLoT | MgCl_2_ 5 mM | 9.25 ±1.70 |
|  |  | MgCl_2_ 8 mM | 26.80 ±0.85 |
|  | dsDNA | MgCl_2_ 5 mM | 9.23 ±0.17 |
|  |  | MgCl_2_ 8 mM | 28.90 ± 1.59 |
| Supplementary  Figure S5B | dsDNA | Urea 0.0 M | 30.06 ±1.68 |
|  |  | Urea 0.2 M | 25.08 ± 0.80 |
|  |  | Urea 0.4 M | 23.91 ± 0.81 |
|  |  | Urea 0.6 M | 16.08 ± 0.54 |
|  |  | Urea 0.8 M | 12.76 ± 0.67 |
|  |  | Urea 1.0 M | 1.27 ± 0.11 |
|  | NiLoT | Urea 0.0 M | 27.70 ± 0.58 |
|  |  | Urea 0.2 M | 26.76 ± 0.90 |
|  |  | Urea 0.4 M | 23.18 ± 0.45 |
|  |  | Urea 0.6 M | 15.19 ± 0.29 |
|  |  | Urea 0.8 M | 12.44 ± 0.58 |
|  |  | Urea 1.0 M | 1.21 ± 0.03 |
| Figure 4A | PCR-based | dsDNA | 20.43 ± 1.57 |
|  |  | NiLoT | 17.70 ± 1.09 |
|  | Plasmid-based | dsDNA | 8.08 ± 0.36 |
|  |  | NiLoT | 9.63 ± 0.62 |
| Figure 4B | 1.0 kb | dsDNA | 9.20 ± 0.42 |
|  |  | NiLoT | 10.75 ± 0.10 |
|  | 3.9 kb | dsDNA | 19.84 ± 0.24 |
|  |  | NiLoT | 19.78 ± 0.56 |
| Figure 4C | WT #1 | dsDNA | 23.39 ± 0.97 |
|  |  | NiLoT | 24.27 ± 0.19 |
|  | WT #2 | dsDNA | 20.84 ± 0.75 |
|  |  | NiLoT | 18.43 ± 0.54 |
|  | mutant #1 | dsDNA | 28.16 ± 0.86 |
|  |  | NiLoT | 26.24 ± 0.63 |
| Figure 4E | Unmodified | dsDNA | 26.39 ± 0.48 |
|  |  | NiLoT | 24.48 ± 0.87 |
|  | Ψ | dsDNA | 22.24 ± 0.99 |
|  |  | NiLoT | 22.06 ± 0.40 |
|  | m^1^Ψ | dsDNA | 22.12 ± 0.96 |
|  |  | NiLoT | 21.87 ± 0.23 |
|  | m^5^C | dsDNA | 22.54 ± 0.96 |
|  |  | NiLoT | 20.53 ± 0.26 |
|  | m^6^A | dsDNA | 18.40 ± 1.05 |
|  |  | NiLoT | 18.48 ± 0.32 |

**Supplementary Table S3.** Kinetic parameters from the exponential equation fitting of DNA–RNA hybrid accumulation. CI; 95% confidence intervals.

| **Parameter** | **dsDNA (Best-fit)** | **dsDNA (95% CI)** | **NiLoT (Best-fit)** | **NiLoT (95% CI)** |
| --- | --- | --- | --- | --- |
| Y₀ | 781 | Not applicable | -581.0 | -1647 to 453 |
| Plateau | ~ 1.54 x10^5^ | Very wide | 9,500.0 | 8123 to 11400 |
| k (min^-1^) | ~ 8.18 x10^-5^ | Very wide | 0.0297 | 0.0184 to 0.0446 |
| Tau (min, 1/k) | ~ 12,200 | Very wide | 33.7 | 22.4 to 54.5 |
| Half-time  (min, ln2/k) | ~ 8,470 | Very wide | 23.3 | 15.5 to 37.8 |
| R² | 0.7160 | - | 0.9252 | - |


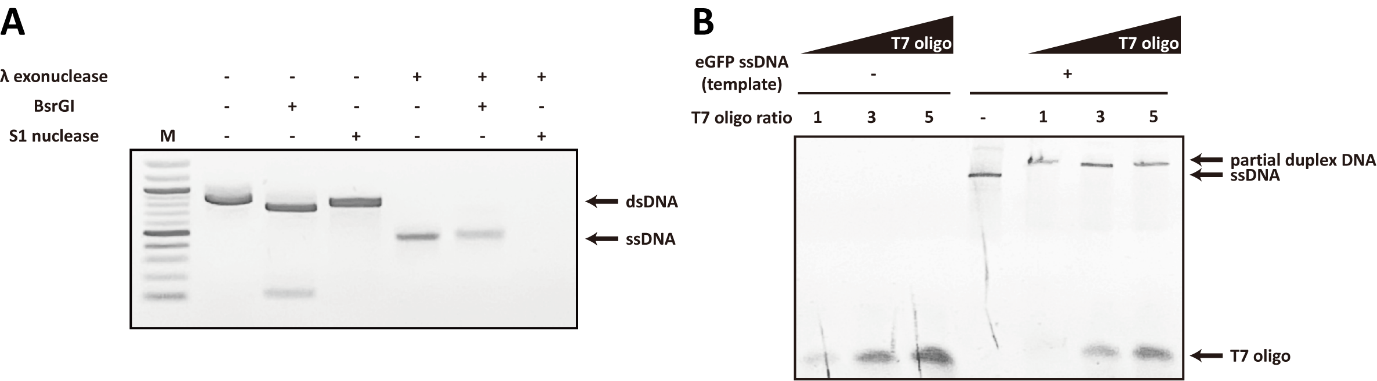


**Supplementary Figure S1.** Generation and validation of partially duplexed DNA templates. (**A**) 1% agarose gel electrophoresis analysis of strand-specific digestion to confirm generation of single-stranded eGFP template strand. Full-length dsDNA was amplified by PCR using a 5′-phosphorylated forward primer, followed by λ exonuclease treatment to selectively degrade the non-phosphorylated reverse strand. Resulting DNA was digested with BsrGΙ (a dsDNA-specific restriction enzyme) or S1 nuclease (specific for single-stranded regions). (**B**) 4% native PAGE analysis of partial duplex template formation by annealing the T7 promoter-containing oligonucleotide (T7 oligo) to the ssDNA template generated in panel (A).


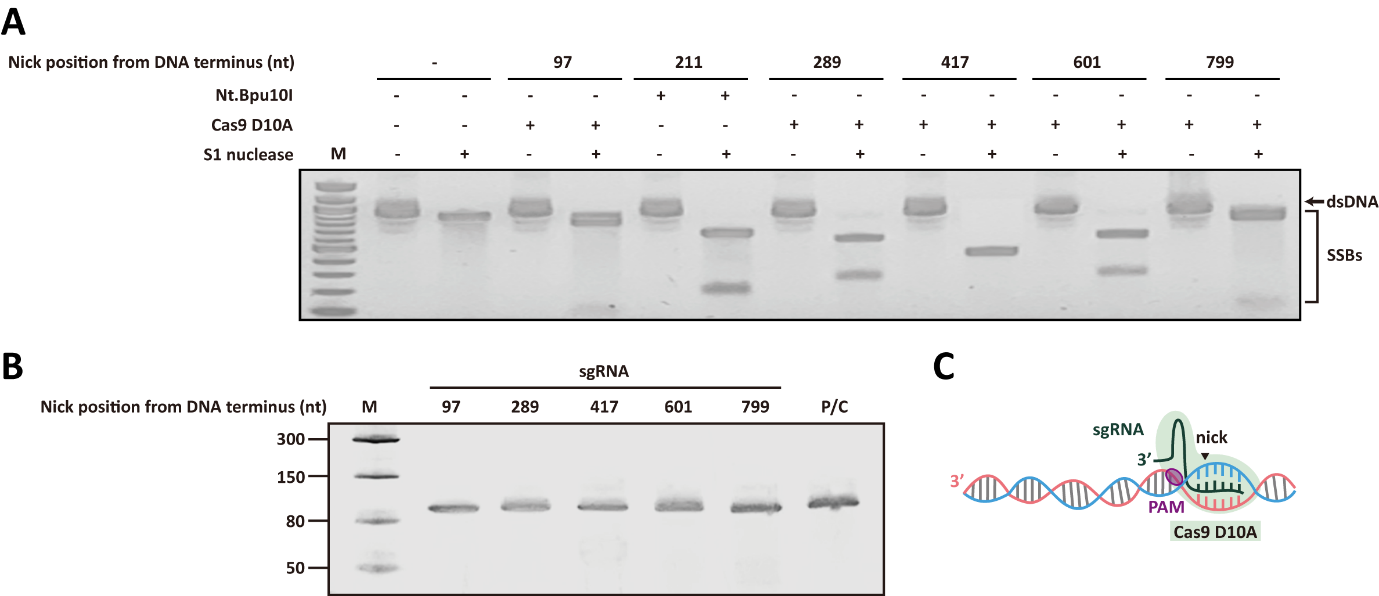


**Supplementary Figure S2.** Verification of site-specific nicking at defined positions on the non-template DNA strand. (**A**) Agarose gel electrophoresis (1%) following S1 nuclease digestion to confirm single-strand break (SSB) formation at specific positions in NiLoT templates. Nicks were introduced into the non-template strand at positions 97, 211, 289, 417, 601, or 799 nt from the 3′ DNA terminus. The 211 nt nick was generated using the strand-specific nicking enzyme Nt.Bpu10I, while the others were introduced using Cas9 D10A with position-specific sgRNAs. Fragmentation patterns are consistent with efficient and accurate SSB induction at the intended sites. (**B**) Denaturing PAGE (10%) analysis of sgRNAs targeting positions 97, 289, 417, 601, and 799 nt. Each sgRNA was resolved to verify integrity and correct size prior to use in Cas9 nicking reactions. (**C**) Schematic representation of site-specific nicking using the Cas9 D10A nickase mutant, which introduces a single-strand break on the non-template strand upon binding to a DNA target specified by a designed sgRNA.


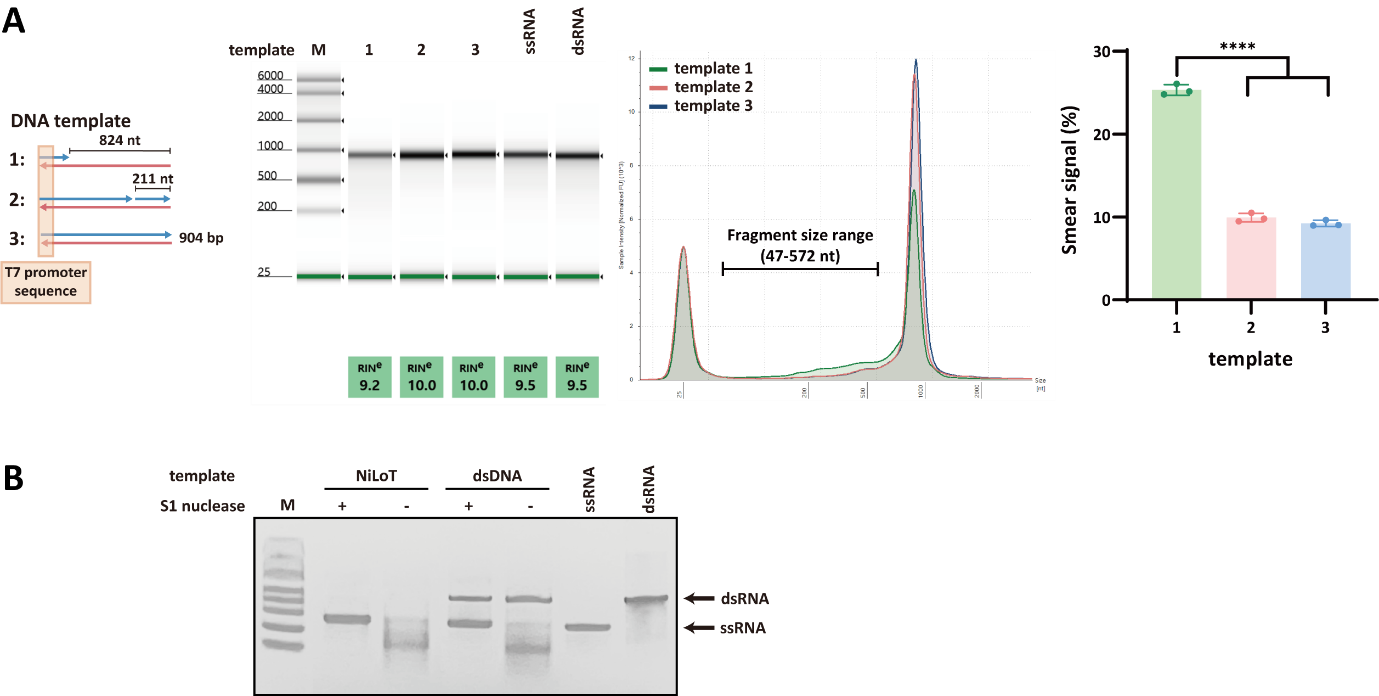


**Supplementary Figure S3.** Integrity and dsRNA analysis of NiLoT-derived IVT RNA. (**A**) TapeStation analysis of RNA transcribed from three DNA templates: partially duplexed DNA (1), nicked DNA (NiLoT, 2), and fully duplexed DNA (3). RNA integrity was evaluated using the RNA Integrity Number equivalent (RINe), a scale from 1 (degraded) to 10 (highly intact), based on electropherogram peak profiles. Smear content was quantified as the proportion of signal within the 47–572 nt range outside the main transcript peak. NiLoT and dsDNA yielded RINe scores of 10.0, while the partial duplex template produced a lower RINe of 9.2 with elevated smear signal (~25%). Statistical comparisons were performed using one-way ANOVA with Šídák’s multiple comparisons test; ****P < 0.0001. (**B**) 1% agarose gel electrophoresis of RNA transcribed from dsDNA or NiLoT templates, followed by S1 nuclease treatment to assess dsRNA content.


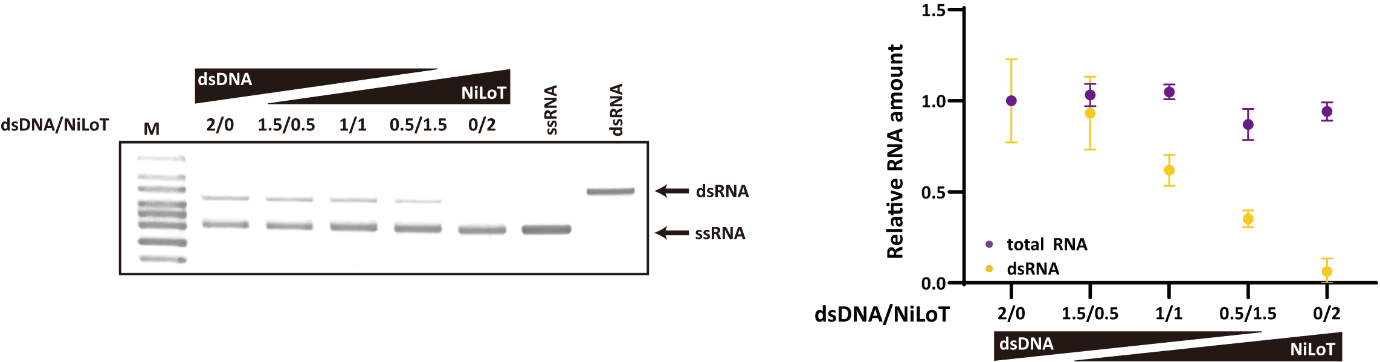


**Supplementary Figure S4.** NiLoT suppresses dsRNA formation in a template ratio-dependent manner. 1% agarose gel electrophoresis of RNA generated from IVT reactions using defined molar mixtures of dsDNA and NiLoT templates. dsRNA levels were quantified based on the gray value intensity of the dsRNA band. Total RNA was quantified using the RiboGreen assay. All values for total RNA and dsRNA were normalized to those obtained from the dsDNA-only template condition.


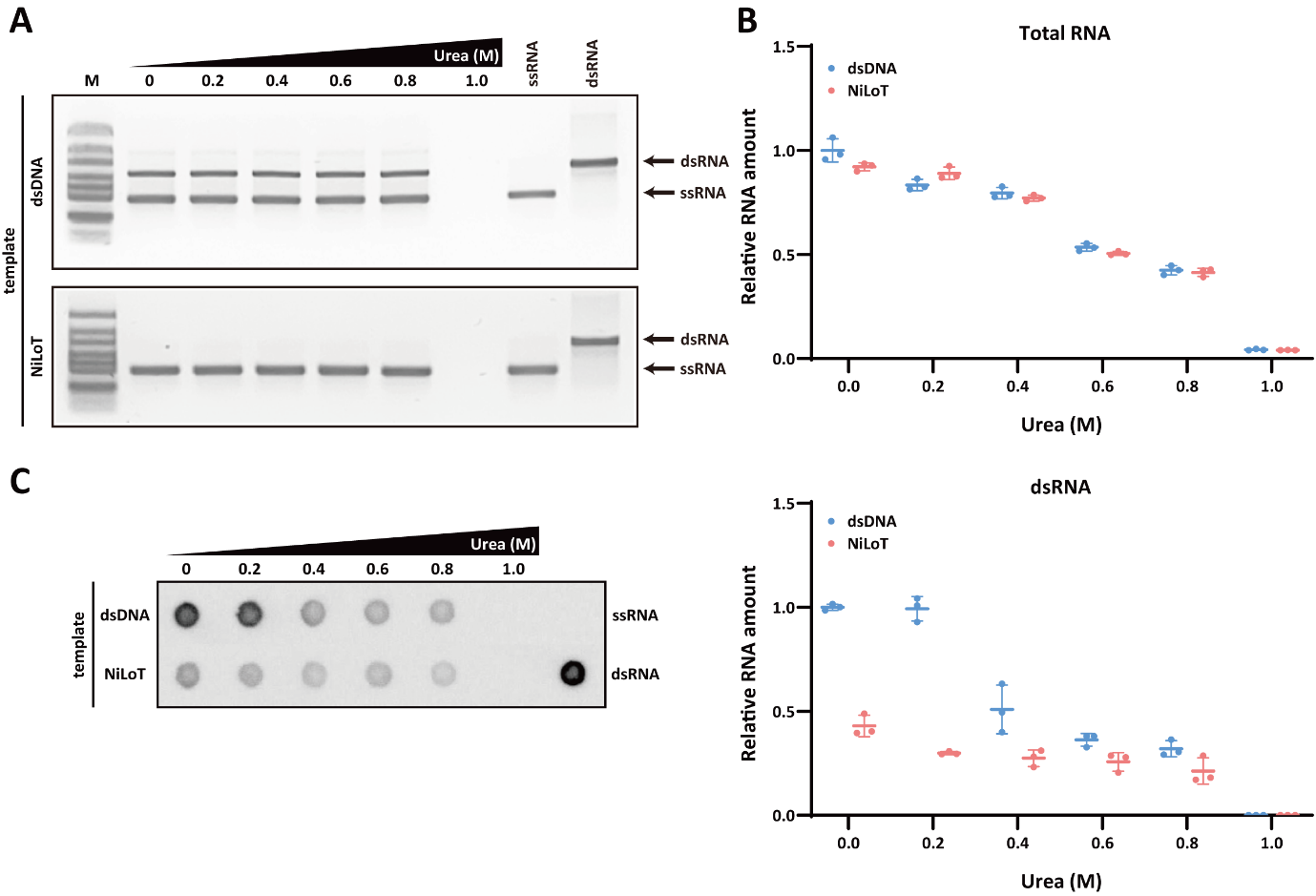


**Supplementary Figure S5.** Comparison of NiLoT and urea-mediated suppression of dsRNA during IVT. (**A**) Agarose gel electrophoresis of IVT RNA synthesized using either a standard dsDNA template or a NiLoT template in the presence of 0–1.0 M urea. A total of 200 ng RNA per lane was loaded. As controls, 200 ng of ssRNA ladder and 20 ng of synthetic dsRNA marker were included to guide size and duplex content. (**B**) RiboGreen quantification of total RNA yield under varying urea concentrations. Data were normalized to the yield obtained from the dsDNA template at 0 M urea. (**C**) Dot blot analysis using J2 monoclonal antibody to detect dsRNA. RNA samples (200 ng per spot) were prepared under the same IVT conditions as in panel A. Control spots included 200 ng of ssRNA and 10 ng of synthetic dsRNA. Quantification was normalized to the dsDNA template condition at 0 M urea.


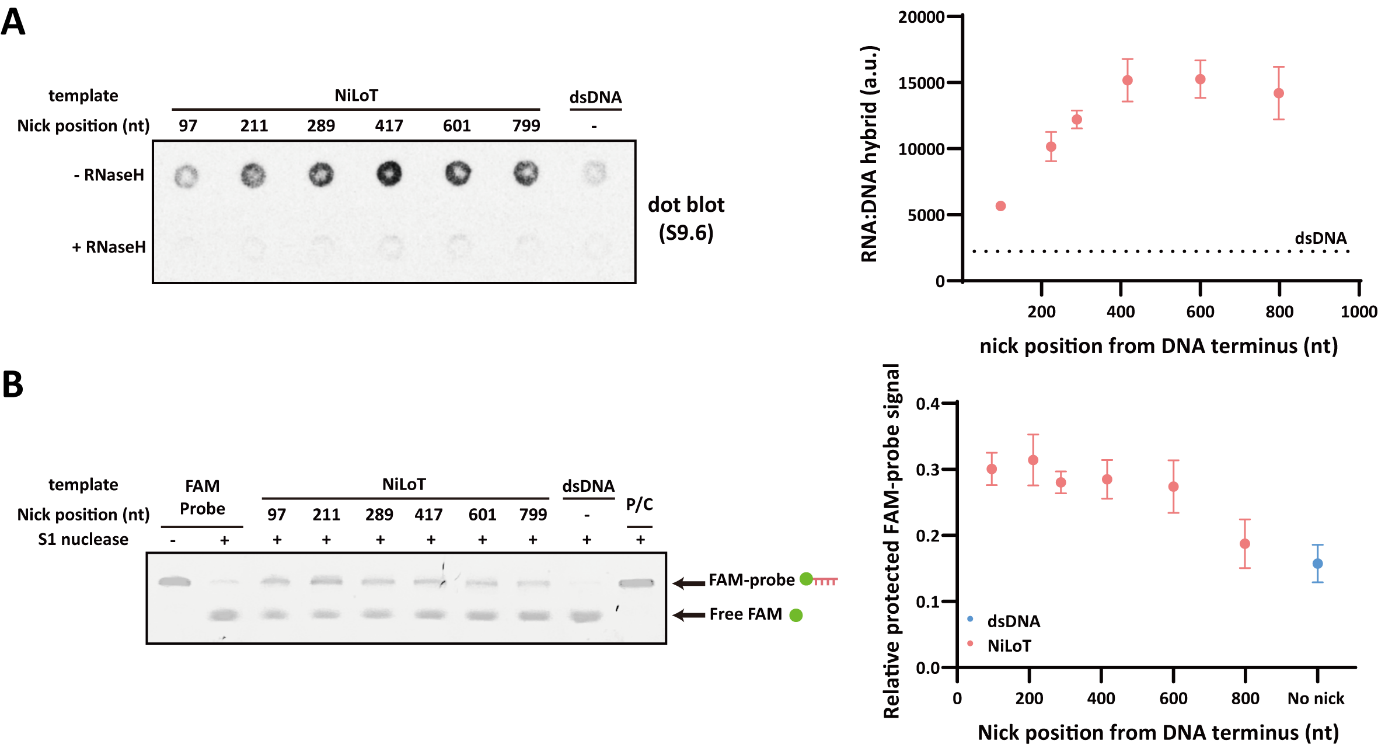


**Supplementary Figure S6.** Distance-dependent decline in strand displacement efficiency with increasing nick distance. (**A**) Dot blot analysis using the S9.6 antibody to detect RNA:DNA hybrids in IVT RNA synthesized from dsDNA or NiLoT templates containing single nicks positioned at varying distances (97–799 nt) from the 3′ end of the non-template strand. Hybrid signal intensity increased with nick distance up to ~400 nt, then plateaued or slightly declined, consistent with a mechanistic threshold for stable R-loop propagation. (**B**) 10% denaturing PAGE analysis of IVT products generated using the same set of templates, followed by hybridization with a 30 nt FAM-labeled probe targeting the displaced non-template strand and subsequent S1 nuclease digestion. P/C: Positive control, generated by pre-annealing the probe with a fully complementary 30 nt oligonucleotide prior to S1 digestion. Band intensities of the protected FAM-labeled probe were quantified and normalized to the positive control. The observed decline in protection signal beyond ~400 nt supports reduced accessibility or reannealing of displaced DNA.


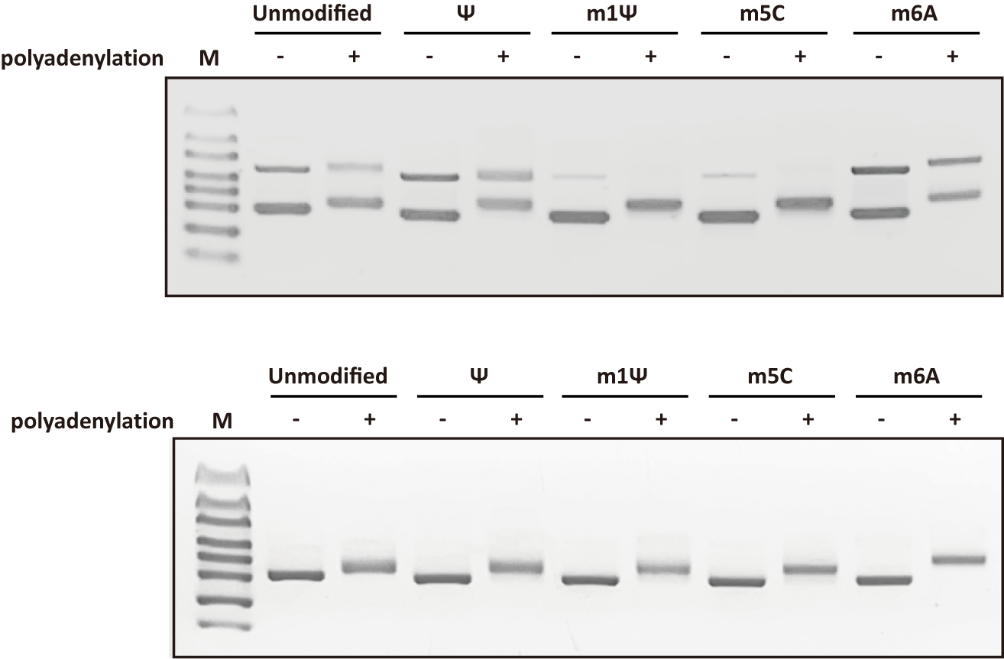


**Supplementary Figure S7.** Evaluation of polyadenylation efficiency following NiLoT. 1% agarose gel electrophoresis analysis of in vitro transcribed RNA synthesized from either dsDNA (top) or NiLoT (bottom) templates with or without modified NTPs, followed by enzymatic polyadenylation.


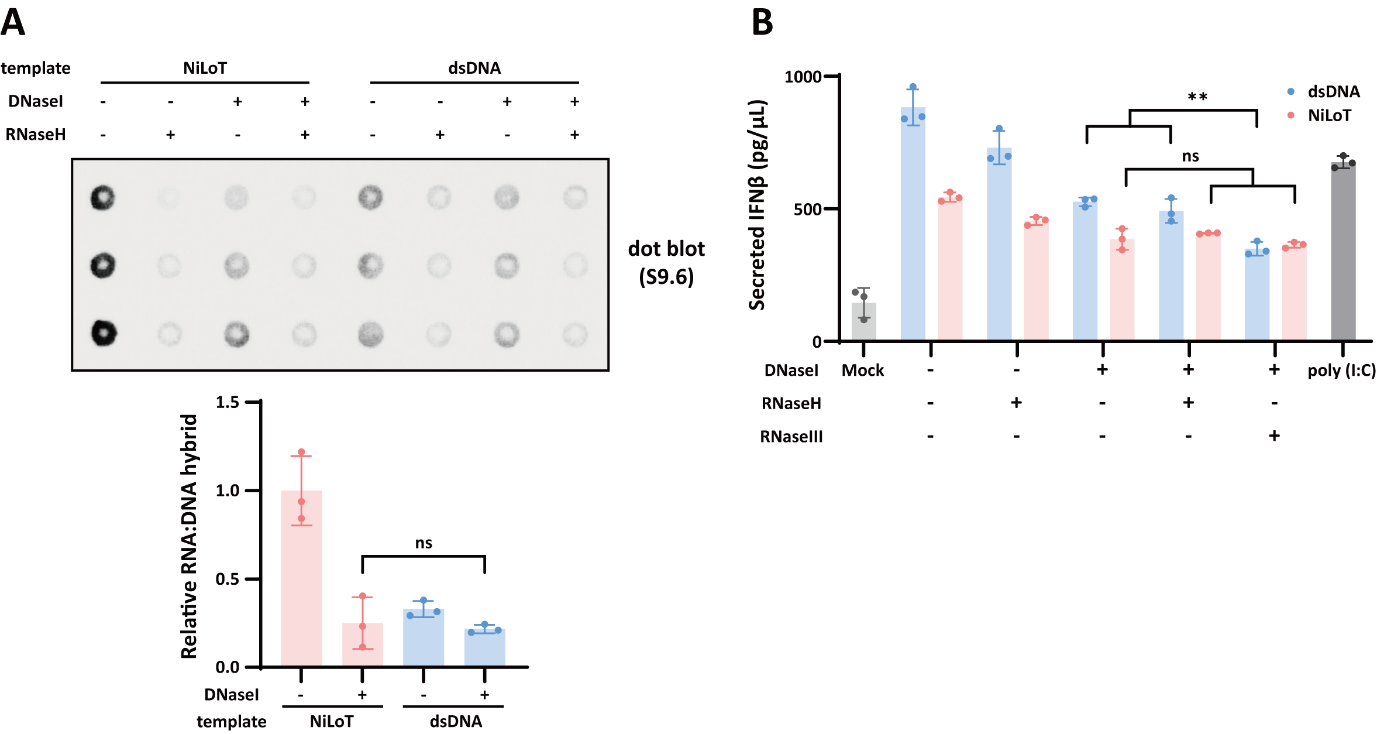


**Supplementary Figure S8.** Characterization of RNA:DNA hybrids and their contribution to innate immune activation. (**A**) Dot blot analysis of RNA:DNA hybrid content in IVT RNA derived from dsDNA or NiLoT templates, using the S9.6 monoclonal antibody after DNase I treatment. Statistical comparisons were made using a two-tailed, unpaired *t*-test; ns = not significant. (**B**) Quantification of IFN-β secretion by ELISA in THP-1 cells transfected with RNAs synthesized from dsDNA or NiLoT templates and subjected to the following nuclease treatments prior to transfection: (i) RNase H^–^/DNase Ι^–^, (ii) RNase H^+^/DNase Ι^–^, (iii) RNase H^–^/DNase Ι^+^, (iv) RNase H^+^/DNase Ι^+^, (v) RNase H^–^/DNase Ι^+^/RNase ΙΙΙ^+^. Cells transfected with Lipofectamine 3000 alone were used as mock controls, and cells treated with poly(I:C) served as positive controls for innate immune activation. Statistical comparisons were performed using one-way ANOVA with Šídák’s multiple comparisons test; **P < 0.01 and ns = not significant.
